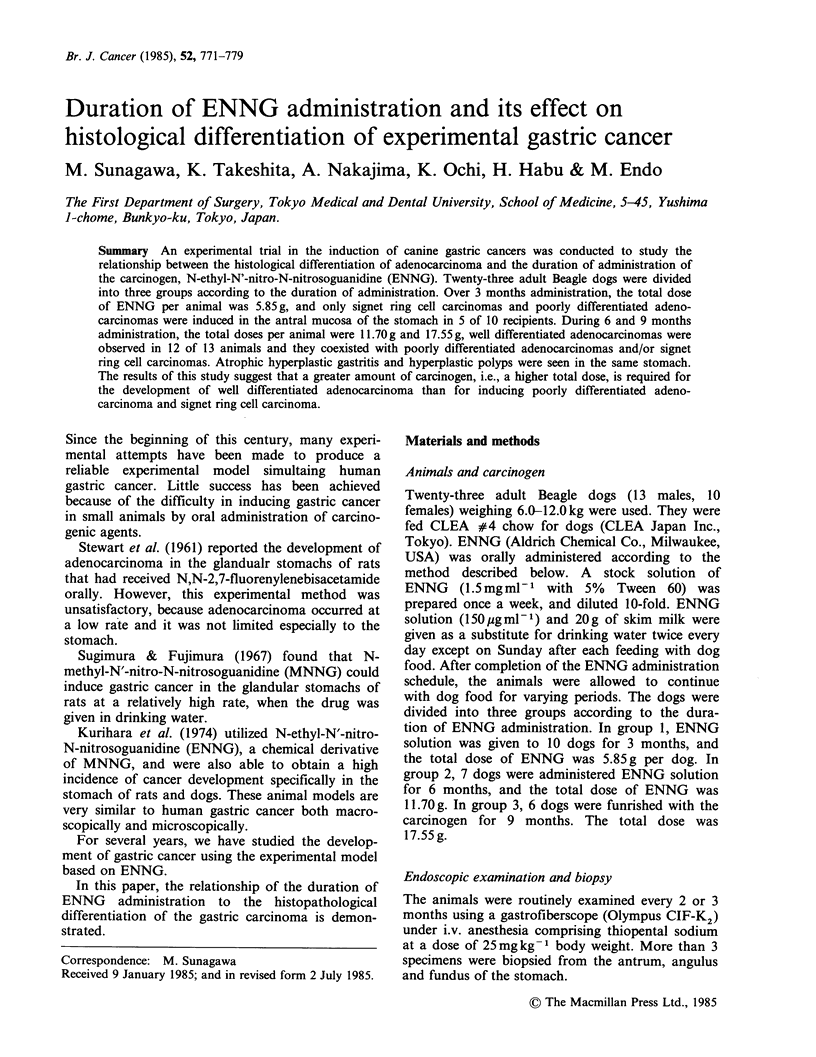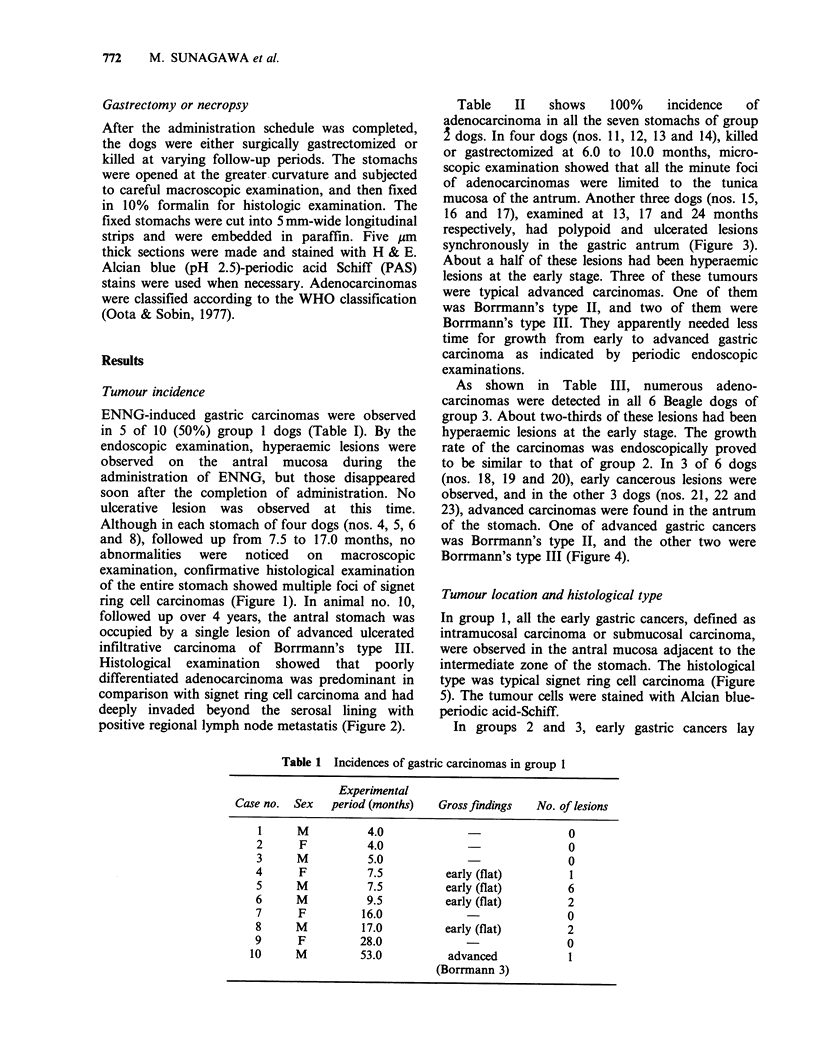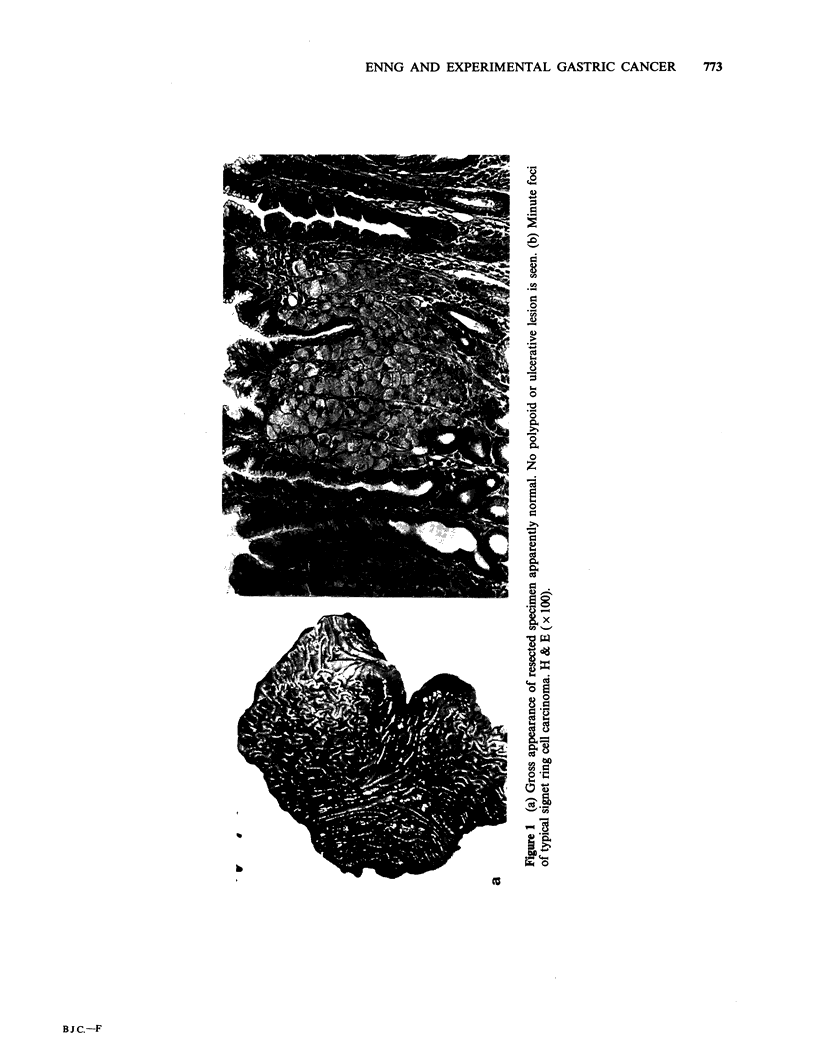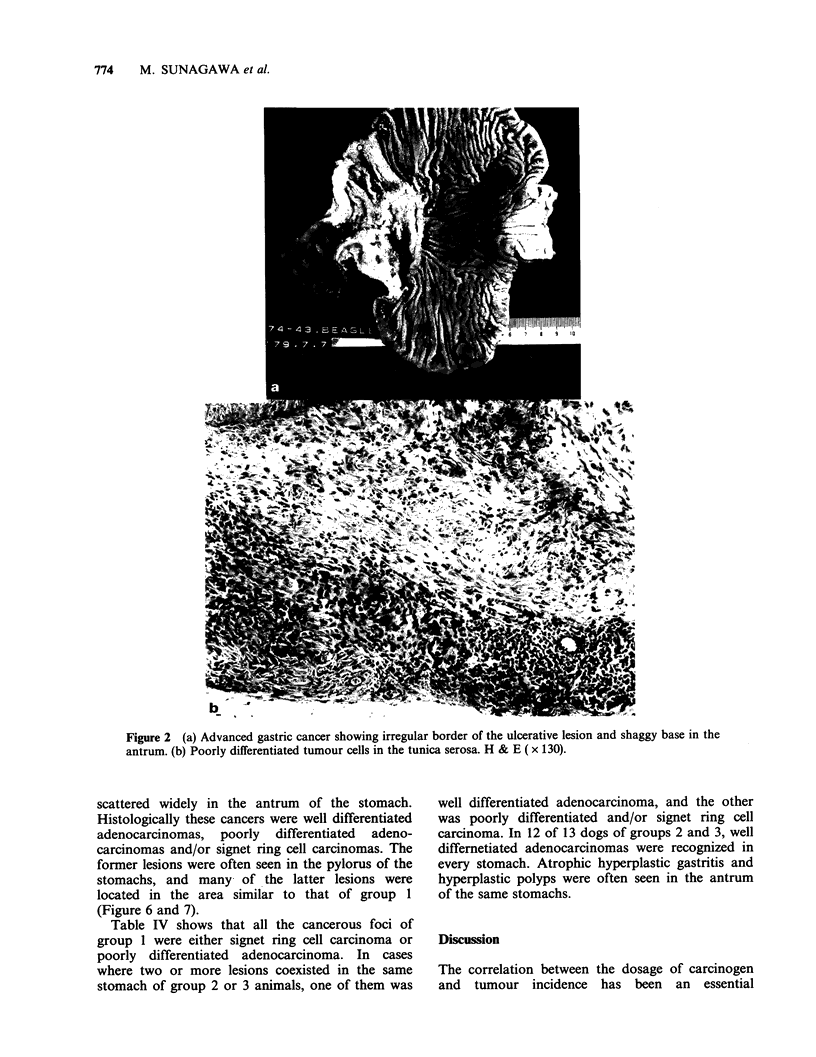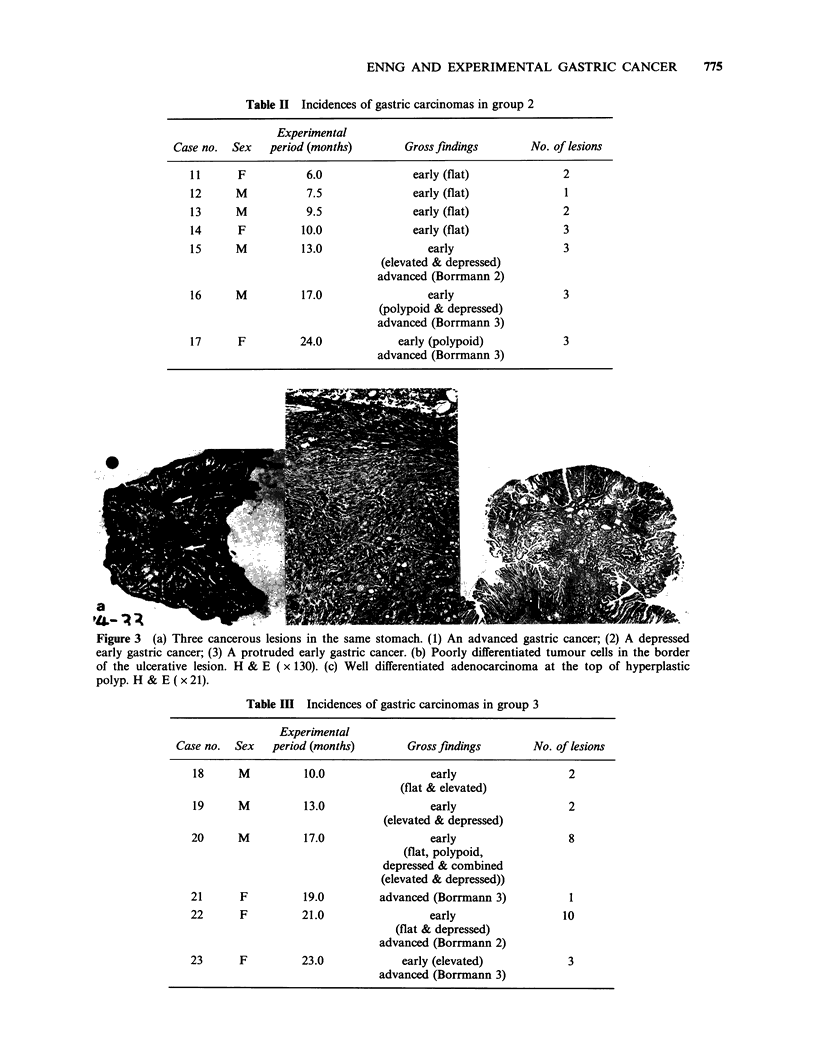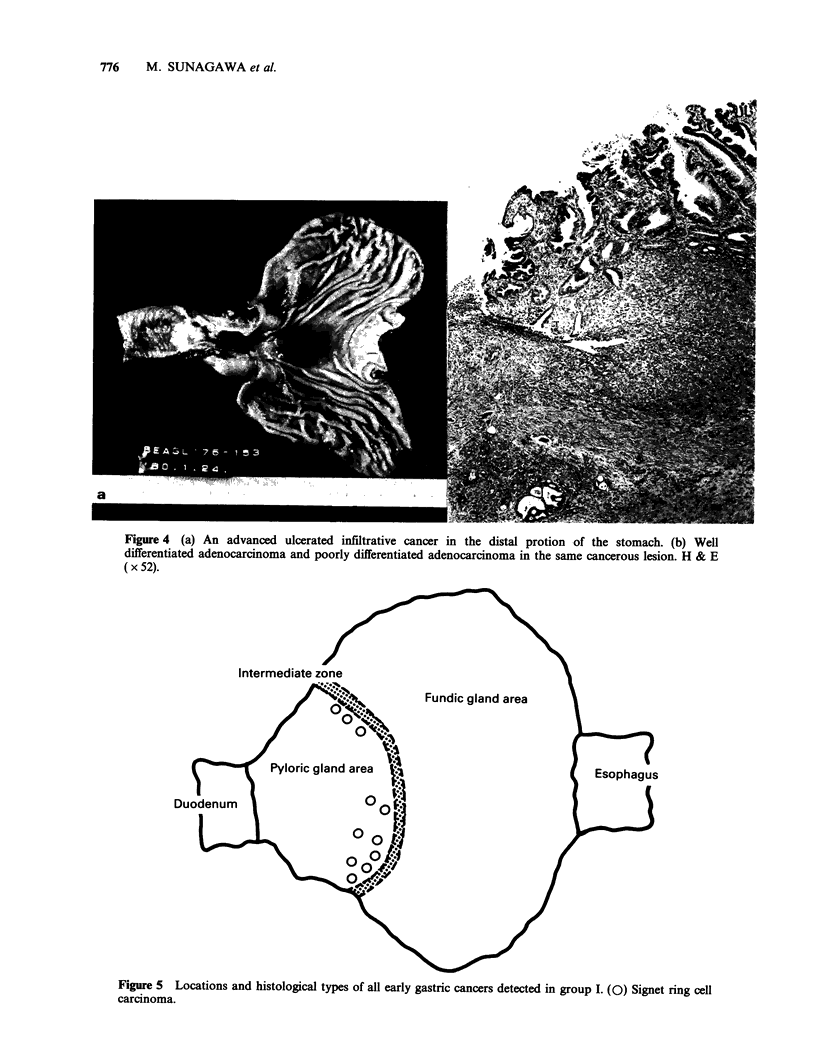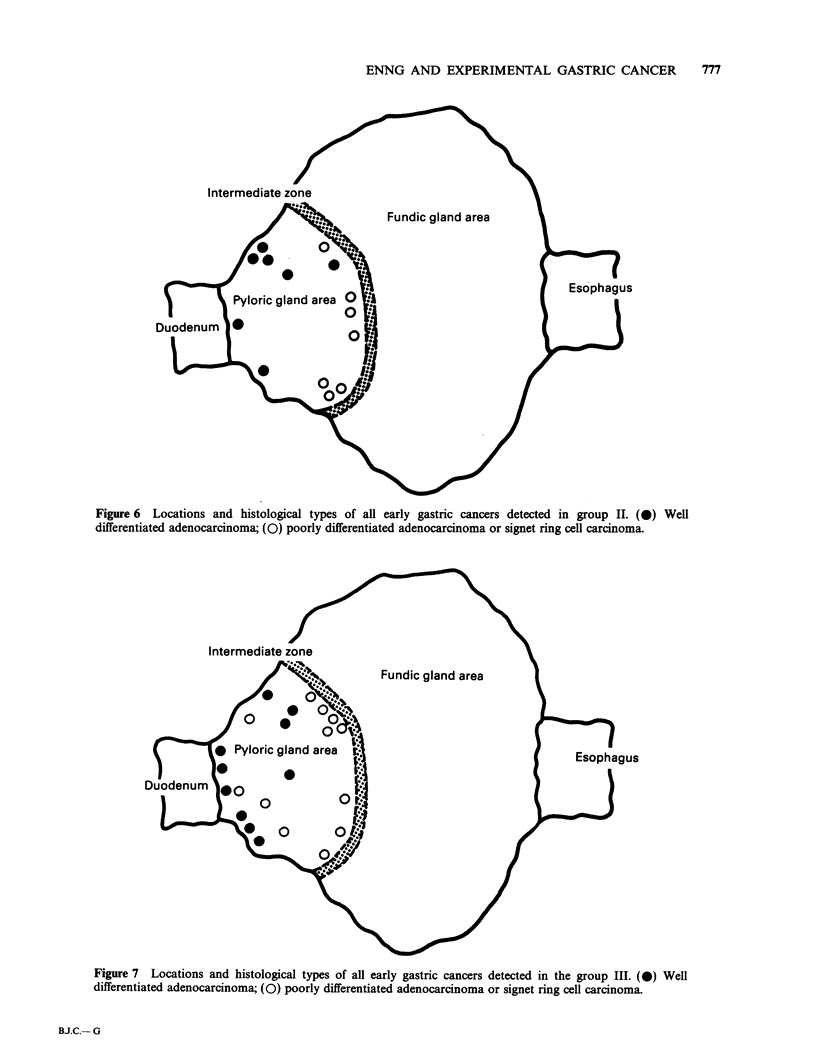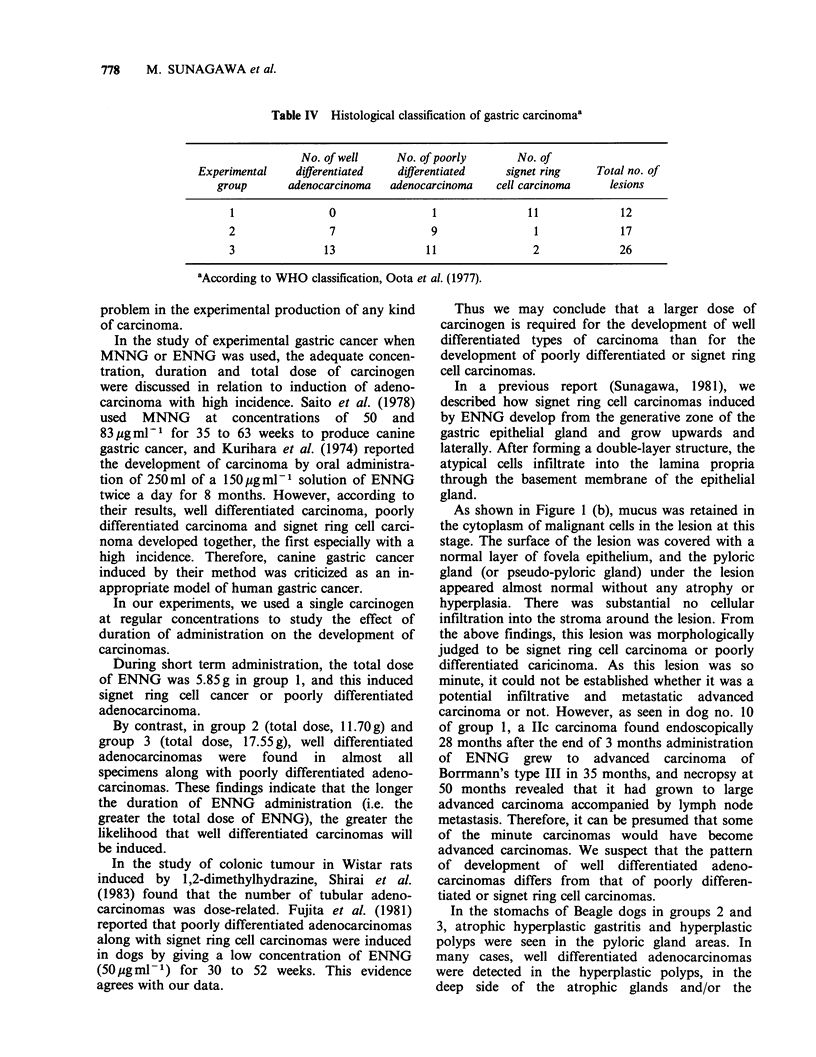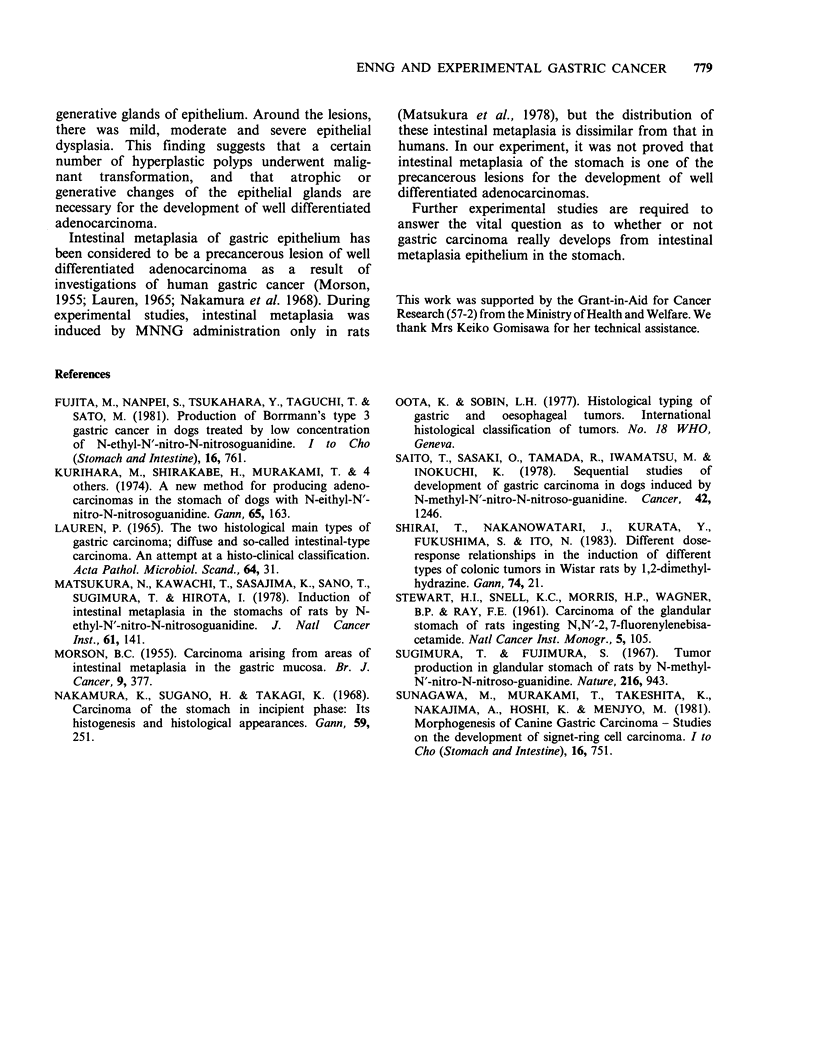# Duration of ENNG administration and its effect on histological differentiation of experimental gastric cancer.

**DOI:** 10.1038/bjc.1985.256

**Published:** 1985-11

**Authors:** M. Sunagawa, K. Takeshita, A. Nakajima, K. Ochi, H. Habu, M. Endo

## Abstract

**Images:**


*Br. J. Cancer* (1985), **52**, 771–779

### Duration of ENNG administration and its effect on histological differentiation of experimental gastric cancer

M. Sunagawa, K. Takeshita, A. Nakajima, K. Ochi, H. Habu & M. Endo

*The First Department of Surgery, Tokyo Medical and Dental University, School of Medicine, 5–45, Yushima 1-chome, Bunkyo-ku, Tokyo, Japan.*

**Summary** An experimental trial in the induction of canine gastric cancers was conducted to study the relationship between the histological differentiation of adenocarcinoma and the duration of administration of the carcinogen, N-ethyl-N'-nitro-N-nitrosoguanidine (ENNG). Twenty-three adult Beagle dogs were divided into three groups according to the duration of administration. Over 3 months administration, the total dose of ENNG per animal was 5.85 g, and only signet ring cell carcinomas and poorly differentiated adenocarcinomas were induced in the antral mucosa of the stomach in 5 of 10 recipients. During 6 and 9 months administration, the total doses per animal were 11.70 g and 17.55 g, well differentiated adenocarcinomas were observed in 12 of 13 animals and they coexisted with poorly differentiated adenocarcinomas and/or signet ring cell carcinomas. Atrophic hyperplastic gastritis and hyperplastic polyps were seen in the same stomach. The results of this study suggest that a greater amount of carcinogen, i.e., a higher total dose, is required for the development of well differentiated adenocarcinoma than for inducing poorly differentiated adenocarcinoma and signet ring cell carcinoma.

Since the beginning of this century, many experimental attempts have been made to produce a reliable experimental model simultaing human gastric cancer. Little success has been achieved because of the difficulty in inducing gastric cancer in small animals by oral administration of carcinogenic agents.

Stewart *et al.* (1961) reported the development of adenocarcinoma in the glandualr stomachs of rats that had received N,N-2,7-fluorenylenebisacetamide orally. However, this experimental method was unsatisfactory, because adenocarcinoma occurred at a low rate and it was not limited especially to the stomach.

Sugimura & Fujimura (1967) found that N-methyl-N′-nitro-N-nitrosoguanidine (MNNG) could induce gastric cancer in the glandular stomachs of rats at a relatively high rate, when the drug was given in drinking water.

Kurihara *et al.* (1974) utilized N-ethyl-N′-nitro-N-nitrosoguanidine (ENNG), a chemical derivative of MNNG, and were also able to obtain a high incidence of cancer development specifically in the stomach of rats and dogs. These animal models are very similar to human gastric cancer both macroscopically and microscopically.

For several years, we have studied the development of gastric cancer using the experimental model based on ENNG.

In this paper, the relationship of the duration of ENNG administration to the histopathological differentiation of the gastric carcinoma is demonstrated.

Correspondence: M. Sunagawa
Received 9 January 1985; and in revised form 2 July 1985.

### Materials and methods

### *Animals and carcinogen*

Twenty-three adult Beagle dogs (13 males, 10 females) weighing 6.0–12.0 kg were used. They were fed CLEA #4 chow for dogs (CLEA Japan Inc., Tokyo). ENNG (Aldrich Chemical Co., Milwaukee, USA) was orally administered according to the method described below. A stock solution of ENNG (1.5 $mg\,ml^{-1}$ with 5% Tween 60) was prepared once a week, and diluted 10-fold. ENNG solution (150 $\mu g\,ml^{-1}$) and 20 g of skim milk were given as a substitute for drinking water twice every day except on Sunday after each feeding with dog food. After completion of the ENNG administration schedule, the animals were allowed to continue with dog food for varying periods. The dogs were divided into three groups according to the duration of ENNG administration. In group 1, ENNG solution was given to 10 dogs for 3 months, and the total dose of ENNG was 5.85 g per dog. In group 2, 7 dogs were administered ENNG solution for 6 months, and the total dose of ENNG was 11.70 g. In group 3, 6 dogs were funrished with the carcinogen for 9 months. The total dose was 17.55 g.

### *Endoscopic examination and biopsy*

The animals were routinely examined every 2 or 3 months using a gastrofiberscope (Olympus CIF-$K_2$) under i.v. anesthesia comprising thiopental sodium at a dose of 25 $mg\,kg^{-1}$ body weight. More than 3 specimens were biopsied from the antrum, angulus and fundus of the stomach.

© The Macmillan Press Ltd., 1985

*Gastrectomy or necropsy*

After the administration schedule was completed, the dogs were either surgically gastrectomized or killed at varying follow-up periods. The stomachs were opened at the greater curvature and subjected to careful macroscopic examination, and then fixed in 10% formalin for histologic examination. The fixed stomachs were cut into 5 mm-wide longitudinal strips and were embedded in paraffin. Five μm thick sections were made and stained with H & E. Alcian blue (pH 2.5)-periodic acid Schiff (PAS) stains were used when necessary. Adenocarcinomas were classified according to the WHO classification (Oota & Sobin, 1977).

### Results

*Tumour incidence*

ENNG-induced gastric carcinomas were observed in 5 of 10 (50%) group 1 dogs (Table I). By the endoscopic examination, hyperaemic lesions were observed on the antral mucosa during the administration of ENNG, but those disappeared soon after the completion of administration. No ulcerative lesion was observed at this time. Although in each stomach of four dogs (nos. 4, 5, 6 and 8), followed up from 7.5 to 17.0 months, no abnormalities were noticed on macroscopic examination, confirmative histological examination of the entire stomach showed multiple foci of signet ring cell carcinomas (Figure 1). In animal no. 10, followed up over 4 years, the antral stomach was occupied by a single lesion of advanced ulcerated infiltrative carcinoma of Borrmann's type III. Histological examination showed that poorly differentiated adenocarcinoma was predominant in comparison with signet ring cell carcinoma and had deeply invaded beyond the serosal lining with positive regional lymph node metastatis (Figure 2).

Table II shows 100% incidence of adenocarcinoma in all the seven stomachs of group 2 dogs. In four dogs (nos. 11, 12, 13 and 14), killed or gastrectomized at 6.0 to 10.0 months, microscopic examination showed that all the minute foci of adenocarcinomas were limited to the tunica mucosa of the antrum. Another three dogs (nos. 15, 16 and 17), examined at 13, 17 and 24 months respectively, had polypoid and ulcerated lesions synchronously in the gastric antrum (Figure 3). About a half of these lesions had been hyperaemic lesions at the early stage. Three of these tumours were typical advanced carcinomas. One of them was Borrmann's type II, and two of them were Borrmann's type III. They apparently needed less time for growth from early to advanced gastric carcinoma as indicated by periodic endoscopic examinations.

As shown in Table III, numerous adenocarcinomas were detected in all 6 Beagle dogs of group 3. About two-thirds of these lesions had been hyperaemic lesions at the early stage. The growth rate of the carcinomas was endoscopically proved to be similar to that of group 2. In 3 of 6 dogs (nos. 18, 19 and 20), early cancerous lesions were observed, and in the other 3 dogs (nos. 21, 22 and 23), advanced carcinomas were found in the antrum of the stomach. One of advanced gastric cancers was Borrmann's type II, and the other two were Borrmann's type III (Figure 4).

*Tumour location and histological type*

In group 1, all the early gastric cancers, defined as intramucosal carcinoma or submucosal carcinoma, were observed in the antral mucosa adjacent to the intermediate zone of the stomach. The histological type was typical signet ring cell carcinoma (Figure 5). The tumour cells were stained with Alcian blue-periodic acid-Schiff.

In groups 2 and 3, early gastric cancers lay

**Table 1** Incidences of gastric carcinomas in group 1

| *Case no.* | *Sex* | *Experimental period (months)* | *Gross findings* | *No. of lesions* |
|---|---|---|---|---|
| 1 | M | 4.0 | — | 0 |
| 2 | F | 4.0 | — | 0 |
| 3 | M | 5.0 | — | 0 |
| 4 | F | 7.5 | early (flat) | 1 |
| 5 | M | 7.5 | early (flat) | 6 |
| 6 | M | 9.5 | early (flat) | 2 |
| 7 | F | 16.0 | — | 0 |
| 8 | M | 17.0 | early (flat) | 2 |
| 9 | F | 28.0 | — | 0 |
| 10 | M | 53.0 | advanced (Borrmann 3) | 1 |

**Figure 1** (a) Gross appearance of resected specimen apparently normal. No polypoid or ulcerative lesion is seen. (b) Minute foci of typical signet ring cell carcinoma. H & E (×100).

**Figure 2** (a) Advanced gastric cancer showing irregular border of the ulcerative lesion and shaggy base in the antrum. (b) Poorly differentiated tumour cells in the tunica serosa. H & E ( × 130).

scattered widely in the antrum of the stomach. Histologically these cancers were well differentiated adenocarcinomas, poorly differentiated adenocarcinomas and/or signet ring cell carcinomas. The former lesions were often seen in the pylorus of the stomachs, and many of the latter lesions were located in the area similar to that of group 1 (Figure 6 and 7).

Table IV shows that all the cancerous foci of group 1 were either signet ring cell carcinoma or poorly differentiated adenocarcinoma. In cases where two or more lesions coexisted in the same stomach of group 2 or 3 animals, one of them was well differentiated adenocarcinoma, and the other was poorly differentiated and/or signet ring cell carcinoma. In 12 of 13 dogs of groups 2 and 3, well differnetiated adenocarcinomas were recognized in every stomach. Atrophic hyperplastic gastritis and hyperplastic polyps were often seen in the antrum of the same stomachs.

### Discussion

The correlation between the dosage of carcinogen and tumour incidence has been an essential

**Table II** Incidences of gastric carcinomas in group 2

| *Case no.* | *Sex* | *Experimental period (months)* | *Gross findings* | *No. of lesions* |
|---|---|---|---|---|
| 11 | F | 6.0 | early (flat) | 2 |
| 12 | M | 7.5 | early (flat) | 1 |
| 13 | M | 9.5 | early (flat) | 2 |
| 14 | F | 10.0 | early (flat) | 3 |
| 15 | M | 13.0 | early<br>(elevated & depressed)<br>advanced (Borrmann 2) | 3 |
| 16 | M | 17.0 | early<br>(polypoid & depressed)<br>advanced (Borrmann 3) | 3 |
| 17 | F | 24.0 | early (polypoid)<br>advanced (Borrmann 3) | 3 |

**Figure 3** (a) Three cancerous lesions in the same stomach. (1) An advanced gastric cancer; (2) A depressed early gastric cancer; (3) A protruded early gastric cancer. (b) Poorly differentiated tumour cells in the border of the ulcerative lesion. H & E ( × 130). (c) Well differentiated adenocarcinoma at the top of hyperplastic polyp. H & E ( × 21).

**Table III** Incidences of gastric carcinomas in group 3

| *Case no.* | *Sex* | *Experimental period (months)* | *Gross findings* | *No. of lesions* |
|---|---|---|---|---|
| 18 | M | 10.0 | early<br>(flat & elevated) | 2 |
| 19 | M | 13.0 | early<br>(elevated & depressed) | 2 |
| 20 | M | 17.0 | early<br>(flat, polypoid,<br>depressed & combined<br>(elevated & depressed)) | 8 |
| 21 | F | 19.0 | advanced (Borrmann 3) | 1 |
| 22 | F | 21.0 | early<br>(flat & depressed)<br>advanced (Borrmann 2) | 10 |
| 23 | F | 23.0 | early (elevated)<br>advanced (Borrmann 3) | 3 |

**Figure 4** (a) An advanced ulcerated infiltrative cancer in the distal protion of the stomach. (b) Well differentiated adenocarcinoma and poorly differentiated adenocarcinoma in the same cancerous lesion. H & E (×52).

**Figure 5** Locations and histological types of all early gastric cancers detected in group I. (○) Signet ring cell carcinoma.

**Figure 6** Locations and histological types of all early gastric cancers detected in group II. (●) Well differentiated adenocarcinoma; (○) poorly differentiated adenocarcinoma or signet ring cell carcinoma.

**Figure 7** Locations and histological types of all early gastric cancers detected in the group III. (●) Well differentiated adenocarcinoma; (○) poorly differentiated adenocarcinoma or signet ring cell carcinoma.

**Table IV** Histological classification of gastric carcinoma[a]

| *Experimental group* | *No. of well differentiated adenocarcinoma* | *No. of poorly differentiated adenocarcinoma* | *No. of signet ring cell carcinoma* | *Total no. of lesions* |
|---|---|---|---|---|
| 1 | 0 | 1 | 11 | 12 |
| 2 | 7 | 9 | 1 | 17 |
| 3 | 13 | 11 | 2 | 26 |

[a]According to WHO classification, Oota *et al.* (1977).

problem in the experimental production of any kind of carcinoma.

In the study of experimental gastric cancer when MNNG or ENNG was used, the adequate concentration, duration and total dose of carcinogen were discussed in relation to induction of adenocarcinoma with high incidence. Saito *et al.* (1978) used MNNG at concentrations of 50 and 83 $\mu g ml^{-1}$ for 35 to 63 weeks to produce canine gastric cancer, and Kurihara *et al.* (1974) reported the development of carcinoma by oral administration of 250 ml of a 150 $\mu g ml^{-1}$ solution of ENNG twice a day for 8 months. However, according to their results, well differentiated carcinoma, poorly differentiated carcinoma and signet ring cell carcinoma developed together, the first especially with a high incidence. Therefore, canine gastric cancer induced by their method was criticized as an inappropriate model of human gastric cancer.

In our experiments, we used a single carcinogen at regular concentrations to study the effect of duration of administration on the development of carcinomas.

During short term administration, the total dose of ENNG was 5.85 g in group 1, and this induced signet ring cell cancer or poorly differentiated adenocarcinoma.

By contrast, in group 2 (total dose, 11.70 g) and group 3 (total dose, 17.55 g), well differentiated adenocarcinomas were found in almost all specimens along with poorly differentiated adenocarcinomas. These findings indicate that the longer the duration of ENNG administration (i.e. the greater the total dose of ENNG), the greater the likelihood that well differentiated carcinomas will be induced.

In the study of colonic tumour in Wistar rats induced by 1,2-dimethylhydrazine, Shirai *et al.* (1983) found that the number of tubular adenocarcinomas was dose-related. Fujita *et al.* (1981) reported that poorly differentiated adenocarcinomas along with signet ring cell carcinomas were induced in dogs by giving a low concentration of ENNG (50 $\mu g ml^{-1}$) for 30 to 52 weeks. This evidence agrees with our data.

Thus we may conclude that a larger dose of carcinogen is required for the development of well differentiated types of carcinoma than for the development of poorly differentiated or signet ring cell carcinomas.

In a previous report (Sunagawa, 1981), we described how signet ring cell carcinomas induced by ENNG develop from the generative zone of the gastric epithelial gland and grow upwards and laterally. After forming a double-layer structure, the atypical cells infiltrate into the lamina propria through the basement membrane of the epithelial gland.

As shown in Figure 1 (b), mucus was retained in the cytoplasm of malignant cells in the lesion at this stage. The surface of the lesion was covered with a normal layer of fovela epithelium, and the pyloric gland (or pseudo-pyloric gland) under the lesion appeared almost normal without any atrophy or hyperplasia. There was substantial no cellular infiltration into the stroma around the lesion. From the above findings, this lesion was morphologically judged to be signet ring cell carcinoma or poorly differentiated caricinoma. As this lesion was so minute, it could not be established whether it was a potential infiltrative and metastatic advanced carcinoma or not. However, as seen in dog no. 10 of group 1, a IIc carcinoma found endoscopically 28 months after the end of 3 months administration of ENNG grew to advanced carcinoma of Borrmann's type III in 35 months, and necropsy at 50 months revealed that it had grown to large advanced carcinoma accompanied by lymph node metastasis. Therefore, it can be presumed that some of the minute carcinomas would have become advanced carcinomas. We suspect that the pattern of development of well differentiated adenocarcinomas differs from that of poorly differentiated or signet ring cell carcinomas.

In the stomachs of Beagle dogs in groups 2 and 3, atrophic hyperplastic gastritis and hyperplastic polyps were seen in the pyloric gland areas. In many cases, well differentiated adenocarcinomas were detected in the hyperplastic polyps, in the deep side of the atrophic glands and/or the generative glands of epithelium. Around the lesions, there was mild, moderate and severe epithelial dysplasia. This finding suggests that a certain number of hyperplastic polyps underwent malignant transformation, and that atrophic or generative changes of the epithelial glands are necessary for the development of well differentiated adenocarcinoma.

Intestinal metaplasia of gastric epithelium has been considered to be a precancerous lesion of well differentiated adenocarcinoma as a result of investigations of human gastric cancer (Morson, 1955; Lauren, 1965; Nakamura *et al.* 1968). During experimental studies, intestinal metaplasia was induced by MNNG administration only in rats (Matsukura *et al.*, 1978), but the distribution of these intestinal metaplasia is dissimilar from that in humans. In our experiment, it was not proved that intestinal metaplasia of the stomach is one of the precancerous lesions for the development of well differentiated adenocarcinomas.

Further experimental studies are required to answer the vital question as to whether or not gastric carcinoma really develops from intestinal metaplasia epithelium in the stomach.

This work was supported by the Grant-in-Aid for Cancer Research (57-2) from the Ministry of Health and Welfare. We thank Mrs Keiko Gomisawa for her technical assistance.

### References

FUJITA, M., NANPEI, S., TSUKAHARA, Y., TAGUCHI, T. & SATO, M. (1981). Production of Borrmann's type 3 gastric cancer in dogs treated by low concentration of N-ethyl-N′-nitro-N-nitrosoguanidine. *I to Cho (Stomach and Intestine)*, **16**, 761.

KURIHARA, M., SHIRAKABE, H., MURAKAMI, T. & 4 others. (1974). A new method for producing adenocarcinomas in the stomach of dogs with N-eithyl-N′-nitro-N-nitrosoguanidine. *Gann*, **65**, 163.

LAUREN, P. (1965). The two histological main types of gastric carcinoma; diffuse and so-called intestinal-type carcinoma. An attempt at a histo-clinical classification. *Acta Pathol. Microbiol. Scand.*, **64**, 31.

MATSUKURA, N., KAWACHI, T., SASAJIMA, K., SANO, T., SUGIMURA, T. & HIROTA, I. (1978). Induction of intestinal metaplasia in the stomachs of rats by N-ethyl-N′-nitro-N-nitrosoguanidine. *J. Natl Cancer Inst.*, **61**, 141.

MORSON, B.C. (1955). Carcinoma arising from areas of intestinal metaplasia in the gastric mucosa. *Br. J. Cancer*, **9**, 377.

NAKAMURA, K., SUGANO, H. & TAKAGI, K. (1968). Carcinoma of the stomach in incipient phase: Its histogenesis and histological appearances. *Gann*, **59**, 251.

OOTA, K. & SOBIN, L.H. (1977). Histological typing of gastric and oesophageal tumors. International histological classification of tumors. *No. 18 WHO, Geneva.*

SAITO, T., SASAKI, O., TAMADA, R., IWAMATSU, M. & INOKUCHI, K. (1978). Sequential studies of development of gastric carcinoma in dogs induced by N-methyl-N′-nitro-N-nitroso-guanidine. *Cancer*, **42**, 1246.

SHIRAI, T., NAKANOWATARI, J., KURATA, Y., FUKUSHIMA, S. & ITO, N. (1983). Different dose-response relationships in the induction of different types of colonic tumors in Wistar rats by 1,2-dimethylhydrazine. *Gann*, **74**, 21.

STEWART, H.I., SNELL, K.C., MORRIS, H.P., WAGNER, B.P. & RAY, F.E. (1961). Carcinoma of the glandular stomach of rats ingesting N,N′-2, 7-fluorenylenebisacetamide. *Natl Cancer Inst. Monogr.*, **5**, 105.

SUGIMURA, T. & FUJIMURA, S. (1967). Tumor production in glandular stomach of rats by N-methyl-N′-nitro-N-nitroso-guanidine. *Nature*, **216**, 943.

SUNAGAWA, M., MURAKAMI, T., TAKESHITA, K., NAKAJIMA, A., HOSHI, K. & MENJYO, M. (1981). Morphogenesis of Canine Gastric Carcinoma – Studies on the development of signet-ring cell carcinoma. *I to Cho (Stomach and Intestine)*, **16**, 751.